# Comparison among Certain Antiseptics and the Agencies

**Published:** 1918-12

**Authors:** 


### COMPARISON AMONG CERTAIN ANTISEPTICS AND THE AGENCIES

It is difficult to choose among the antiseptics; it is equally difficult to choose between the principle of no antiseptics and the principle of antiseptics. This is equal to saying that antiseptics have a relatively low value. But there are distinct merits among antiseptics.

*Carrel-Dakin*: (1) The one that is most universally useful, excepting under battle conditions, is not an antiseptic alone, but a system of treatment—viz., the Carrel-Dakin. This is the method that is most useful in recessed wounds with a tendency to pooling of wound discharge. The merit of the Carrel-Dakin is perhaps owing quite as much to its prevention of the accumulation of wound secretion by the frequent flooding of the wound, as by the antiseptic property of the fluid. (2) It entails much exact nursing and professional care. (3) The technic is exacting and errors easily creep in. (4) It is not practicable under battle conditions.

*Dichloramin-T Chlorcosane*: (1) The technic is the simplest of all the antiseptics. (2) Dressings are made once a day and it is applicable to battle conditions. (3) No rubber tubing and but a small amount of dressing is used. The wound discharge is light. (4) The dressings do not stick, and are usually painless. (5) There being no water used, there is a minimum chance of cross infection during rush periods. (6) The wound heals rapidly. (7) It does not interfere with suturing of the wound. (8) An objection to dichloramin-T chlorcosane is the difficulty of introducing it sufficiently well into recessed, deep wounds. In these, (9) Carrel-Dakin is better.

*Flavine*: (1) It is non-toxic. (2) Its effects for two days are excellent. (3) It is a good dressing during transportation of cases of delayed primary suture. (4) It stains fabric.

*Alcohol*: (1) Alcohol is a good antiseptic, but it coagulates proteins, hence causes an eschared surface. (2) This property makes it valuable in treating small superficial wounds in preparation for excision and suture.

*Wright's Hypertonic*: (1) Most useful for cleaning up foul wounds—perhaps the best method. (2) As an exclusive method, it soon leaves the tissue too soggy and wet.

*Hot Packs*: (1) In acute infections, there is no single treatment equal to hot packs or hot hypertonic salt soaks. (2) Continued too long, the tissues become too much macerated.

*Bip*: (1) Bip restrains bacterial growth. (2) It deadens pain. (3) It provides the valuable "curtain drainage". (4) It requires infrequent dressing, and is a comfortable dressing in transport. (5) It is applicable to all kinds of wounds. (6) It contains two poisons. (7) It interferes with healing. (8) It is valuable in bone infections. (9) A bipped wound discharges rather less than wounds otherwise treated.

From the foregoing, it is apparent that each method has particular merits, especially applicable to certain phases and certain circumstances. Wounds are no longer treated by formulae. Treatment is directed rather toward meeting the varying indications of phases and circumstances.

